# Relationship between gender role, anger expression, thermal discomfort and sleep onset latency in women

**DOI:** 10.1186/1751-0759-3-11

**Published:** 2009-10-13

**Authors:** Mariella von Arb, Britta Gompper, Andrea H Meyer, Elisabeth Zemp Stutz, Selim Orgül, Josef Flammer, Kurt Kräuchi

**Affiliations:** 1 Thermophysiological Chronobiology, Centre for Chronobiology, Psychiatric University Clinics, Wilhelm Klein Strasse 27, 4025 Basel, Switzerland; 2 Department of Psychology, University of Basel, Missionsstrasse 60/62, 4055 Basel, Switzerland; 3 Institute of Social & Preventive Medicine, University of Basel, Steinengraben 49, 4051 Basel, Switzerland; 4 University Eye Clinic, Mittlere Strasse 91, 4031 Basel, Switzerland

## Abstract

**Background:**

Women with thermal discomfort from cold extremities (hands and feet; TDCE) often suffer from prolonged sleep onset latency (SOL). Suppressed anger could contribute to the genesis of both TDCE and prolonged SOL. The aim of the study was to test the hypothesis whether stereotypic feminine gender socialization (SFGS) is related to anger suppression (experienced anger inwards, Anger-In), which in turn could affect TDCE and SOL.

**Methods:**

148 women, a sub-sample of a larger survey carried out in the Canton Basel-Stadt (Switzerland), sent back detailed postal questionnaires about SOL, TDCE, anger expression (STAXI, state -trait -anger -expression -inventory) and SFGS using a gender power inventory, estimating the degree of gender specific power expression explicitly within women by stereotypic feminine or male attribution. Statistics was performed by path analysis.

**Results:**

A significant direct path was found from stereotypic feminine attribution to Anger-In and prolonged SOL. Additionally, a further indirect path from Anger-In via TDCE to SOL was found. In contrast, stereotypic male attribution was not related to Anger-In but was significantly associated with outwardly expressed anger.

**Limitations:**

Self-reported data, retrospective cross-sectional survey, prospective studies are required including physiological measurements.

**Conclusion:**

Stereotypic feminine gender socialization may play an important determinant for anger suppression, which subsequently can lead to thermal discomfort from cold extremities and prolonged sleep onset latency.

## Background

It is well known that sleep onset latency (SOL) is prolonged in many life circumstances, such as stress, diverse illnesses and exciting behaviors before sleep [1]. Our extremities, hands and feet, are highly sensitive to react with vasoconstriction in these situations [2]. Both phenomenons seem to be closely interrelated. It has recently been published that subjects with prolonged SOL often suffer from thermal discomfort from cold extremities (TDCE) [3]. About 30% of women aged 20-40 years complain from cold hands and feet, but only 7% of men [3]. Independent of gender, each seventh subject complaining of TDCE also exhibits difficulties initiating sleep [3] - the relative risk in these subjects was approximately doubled.

TDCE is closely related to the body's thermophysiology, which can be declared as a state of increased body heat retention [3,4]. Our sleep is typically initiated by body heat loss usually occurring on the declining portion of the CBT curve when its rate of change, and body heat loss, is maximal [5,6]. Before lights off, body heat loss promotes sleepiness and the rapid onset of sleep via distal vasodilatation [3,7,8]. Distal vasodilatation and heat redistribution from the thermal core to the thermal shell seems to represent the main determining thermophysiological components of a well-orchestrated down-regulation of core body temperature in the evening [3]. Distal vasoconstriction and TDCE may therefore have clinical relevance for insomnia, in particular for difficulties initiating sleep.

Besides the many known determinants of TDCE [9], the skin sympathetic nervous system is of importance for vascular reactivity to cold and emotional stimuli [2]. Especially feeling of anger is related to increased vascular reactions [10-16]. These findings suggest that feeling of anger could contribute to the etiology of the TDCE and hence of prolonged SOL. Furthermore, clinical and epidemiological studies have shown that girls are socialized in such a way that their expression of anger and aggression are strongly constrained [17,18]. This socially conform behavior of women could lead to an anger suppression problem (e.g. higher STAXI Anger-In -score; [19,20]. However, there is no study differentiating between women with or without a socially conform feminine behavior with respect to anger suppression and the development of psychosomatic disturbances such as TDCE and prolonged SOL.

Therefore, the aim of the study is to test the hypothesis whether stereotypic feminine gender socialization is related to anger suppression, which in turn could lead to TDCE and prolonged SOL, relying on a path-analysis with an a-priori formulated structure model. A part of the study results has been published in abstract form [21].

## Methods

### Subjects & Procedure

This study is a follow-up study of a large postal questionnaire survey on the relationship between sleep onset behavior and thermal discomfort [3], approved by the ethical committee of the cantons Basel-Stadt and Baselland (EKBB) and carried out between February and May 2006. The survey included 1400 men and 1400 women, aged 20-40 years, a random sample of the population register of Basel-Stadt, Switzerland. Based on the survey's finding that the prevalence of TDCE is 4 to 5 times higher in women than in men [3], a detailed postal questionnaire were sent to those women, who had consented to be re-contacted for a further study (N = 520 of total 1001 women). 148 of the 520 women returned completed questionnaires. They did not statistically differ from the rest of women who participated only in the first study part (N = 853) with respect to their socio- and bio-demographic characteristics shown in Table 1.

**Table 1:** Socio, psycho and bio-demographic characteristics

**Variable**	**Mean ± sem**	**Median (IQR)**
STAXI scores:		
-State Anger	11.87 ± 0.29	11 (2)
-Trait Anger	18.57 ± 0.45	18 (6)
-Anger-In	14.57 ± 0.37	14 (6)
-Anger-Out	12.62 ± 0.29	12 (4)
-Anger-Control	21.85 ± 0.33	22 (6)
SFA	2.25 ± 0.04	2.20 (0.58)
SMA	2.49 ± 0.04	2.55 (0.71)
TDCE	2.29 ± 0.06	2 (1)
BMI	22.21 ± 0.33	21.41 (3.85)
Age (yr)	33.3 ± 0.5	34 (11)
# cigarettes	2.55 ± 0.46	0 (2)
Contraceptives (%)	37	
Hours work/week	30.6 ± 1.1	33.5 (19.0)
Sleep duration (h)	7.8 ± 0.1	7.8 (1.0)
Sleep midpoint (hr)	3.3 ± 0.1	3.3 (1.0)
Sleep onset latency (min)	18.5 ± 1.4	12.5 (25.0)
Time awake/night (min)	24.4 ± 2.5	15.0 (22.5)
# waking-ups/night sleep	1.9 ± 0.2	1.5 (1.0)

SFA = stereotypic feminine attributes; SMA = stereotypic masculine attributes; TDCE = thermal discomfort from cold extremities; BMI = body mass index (kg/m^2^); sleep mid point = [sleep onset time (hr) -sleep offset time (hr) -sleep onset latency (h)]/2, weekly mean. IQR indicates inter- quartile -range of the study sample.

### Instruments

#### Gender- Power- Inventory

Many attributes are clearly assigned to gender [22]. Certain assignments imply additionally a gender specific suppression or expression of power. Previous instruments addressed stereotypic feminine gender socialization [22,23] but did not take into account the important aspect of power suppression or expression implicitly represented in certain attributes. For the present study, a new gender power inventory has been developed (by M.v.A.) estimating the degree of gender specific power suppression and expression explicitly within women. Power suppression can be estimated by specific stereotypic feminine attributes (SFA) and conversely power expression by specific stereotypic male attributes (SMA). Based on this background, twenty-one bipolar items have been chosen, ten items referring to specific power suppression (SFA; "in comparison to other women I'm more.: sensitive, controlled, adjusted, holding back, inhibited, avoiding confrontation, emotional, inferior, anxious, depressed"), and a further eleven items referring to specific power expression (SMA; "in comparison to other women I'm more.: loud, active, vigorous, resolute, impulsive, confident, dominant, forceful, spontaneous, aggressive, quick to make decision) [22-27]. Four answer categories were given: absolutely not the case = 1, rather not the case = 2, rather the case = 3, absolutely the case = 4. A mean SFA and SMA -score has been calculated. With respect to other women, a high SFA -score indicates relative high power suppression and a high SMA -score indicates relative high power expression.

### State-Trait-Anger-eXpression-Inventory (STAXI)

The STAXI [19,20] was used for dispositional state and trait anger, as well as for anger expression. It consists of three different scales, State Anger (10 items), Trait Anger (10 items), and anger expression (24 items). SA refers to the intensity of the individual's angry feelings at the time of testing. TA measures the extent to which an individual is predisposed to experience anger or frustration in a range of situations. Individuals are asked to indicate on a four-point scale how often they generally react or behave in the manner described by each item. Anger expression consists of three subscales. Anger-In measures the extent to which people hold things in or suppress anger when they are angry or furious. Anger-Out describes the extent to which a person expresses her emotional experience of anger in an outwardly manner. Anger-Control involves expenditure of energy to monitor and control the physical or verbal expression of anger. A high score on each of these scales represents a high tendency or frequency to express that mode of anger. The STAXI has demonstrated good internal reliability and validity based on results from a variety of samples and cultures [19,20].

### Thermal discomfort from cold extremities (TDCE)

Two questions referring to the leading symptoms of TDCE were used for its definition: 1. In the past month, how intensively did you suffer from cold hands? 2. In the past month, how intensively did you suffer from cold feet? Answer categories: 0 = 'not at all', 1 = 'a little', 2 = 'quite', 3 = 'extraordinary'. For dimensional analysis a TDCE-score of the two questions was calculated (Σ = 0-6). TDCE has been externally validated with fingertip skin temperature [3].

### Sleep parameters

Sleep onset latency (SOL) was inquired by the question: "During the past month, how long (in minutes) has it usually taken to fall asleep?" Log transformed sleep onset latency [log(SOL)] was utilized for dimensional analysis to obtain normally distributed values. Time awake and number of waking-ups during a night's sleep episode were asked by the questions "In the past month, for how long have you been awake during a night's sleep episode?" and "In the past month, how often did you usually wake-up during a night's sleep episode?", respectively. Sleep times for the night's sleep episode were asked in a similar way e.g.: "In the past month when did you usually switch the lights off?"

### Statistical analyses

Statistical analysis was performed using Statistica 7.0 (StatSoft, Inc., Tulsa, OK 74104, USA). Pearson's correlation analysis was used for bivariate testing. Path analysis was calculated using the statistical package AMOS 5.0.

## Results

### Sample characteristics

Table 1 shows the characteristic of the study sample. Our sub-sample of 148 women didn't statistically differ from women of the large survey carried out in Canton Basel-Stadt [3] with respect to BMI, age, contraceptive prevalence, sleep times, sleep onset latency, hours work/week, number of cigarette consumption and TDCE, indicating a representative sample of the general population of Basel-Stadt.

### Bivariate correlation analysis

Pairwise correlations of the relevant variables are shown in Table 2. SFA is highly negatively correlated with SMA. SFA exhibited positive correlations with Anger-Control and most remarkable and highly significant with AI. SFA also correlated significantly with TDCE and log (SOL), whereas TDCE is positively inter-correlated with log (SOL). SMA is significantly correlated with all three anger expression scores, negatively with Anger-In and Anger-Control and positively with Anger-Out. SMA is negatively correlated with log (SOL). Trait and state anger did not exhibit significant correlations with SFA, SMA, TDCE and log (SOL) (data therefore not shown).

**Table 2:** Inter-correlations (r) of selected variables

	**SFA**	**SMA**	**AI**	**AO**	**AC**	**TDCE**	**logSOL**	**awake**
SFA	1	-0.882***	0.515***	-0.031	0.170*	0.172*	0.218*	0.076
SMA		1	-0.292***	0.292***	-0.240***	-0.181*	-0.068	-0.003
AI			1	-0.023	0.280***	0.196*	0.322***	0.079
AO				1	-0.422***	0.071	-0.041	0.001
AC					1	0.147	0.009	-0.096
TDCE						1	0.235**	0.017
logSOL							1	0.329***
awake								1

Inter-correlation table between selected variables (Pearson's r-correlation values). SFA = stereotypic feminine attributes; SMA = stereotypic male attributes; AI = anger expression inwards; AO = outwardly expressed anger; AC = controlled anger expression; TDCE = thermal discomfort from cold extremities; log (SOL) = log-transformed values of estimated sleep onset latency (min); awake = estimated time awake (min) during a night sleep episode. *p < 0.05, **p < 0.01, ***p < 0.001

Interestingly enough, time awake during the sleep episode (awake in min) neither correlated with any of the anger expression scores nor with SFA and SMA. The three anger expression scores show similar significant inter-correlations as previously published [19,20], confirming the reliable factor structure of the questionnaire in our sample. Among the anger expression scores only Anger-In correlated with log (SOL) (r = 0.322). Furthermore, log (SOL) showed a significant correlation (r = 0.329) with time awake during the sleep episode indicating that sleep onset disturbances are associated with sleep maintenance disturbance.

**Figure 1:**
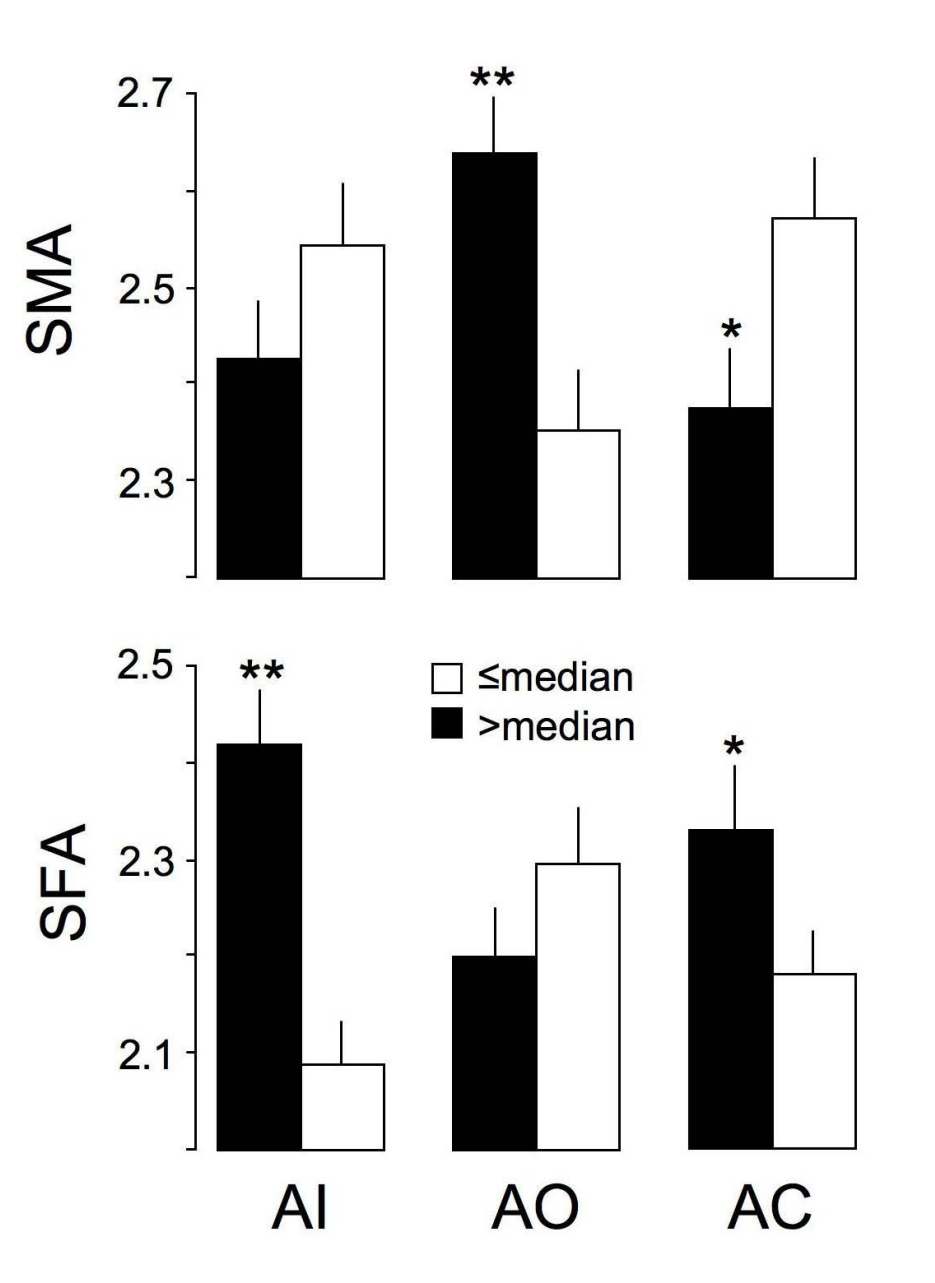
**Stereotypic masculine (upper panel, SMA) or feminine (lower panel, SFA) attributes according to high (beyond median, black column) or low (equal or below median, white column) anger expression categories**. AI = anger inwards, AO = anger out, AC = anger control. * = p < 0.05; ** = p < 0.01 indicate significant differences between median split values (> vs. ≤ median) within AI, AO and AC.

In order to highlight the relationship between anger expression and stereotypic gender attribution, SFA and SMA has been analyzed as a function of high or low level (median splitted -data) of Anger-In, Anger-Control and Anger-Out -scores (Figure 1). Women with high Anger-In score (>median) rated significantly higher SFA than those with low Anger-In score (≤median), but did not differ in SMA. Conversely, women with high Anger-Out -score rated significantly higher SMA than those with low Anger-Out -score, but did not differ in SFA. Anger-Control differentiated regarding to both SFA and SMA: in women with high Anger-Control -score, significantly higher SFA and lower SMA was found, respectively.

### Path-analysis

**Figure 2:**
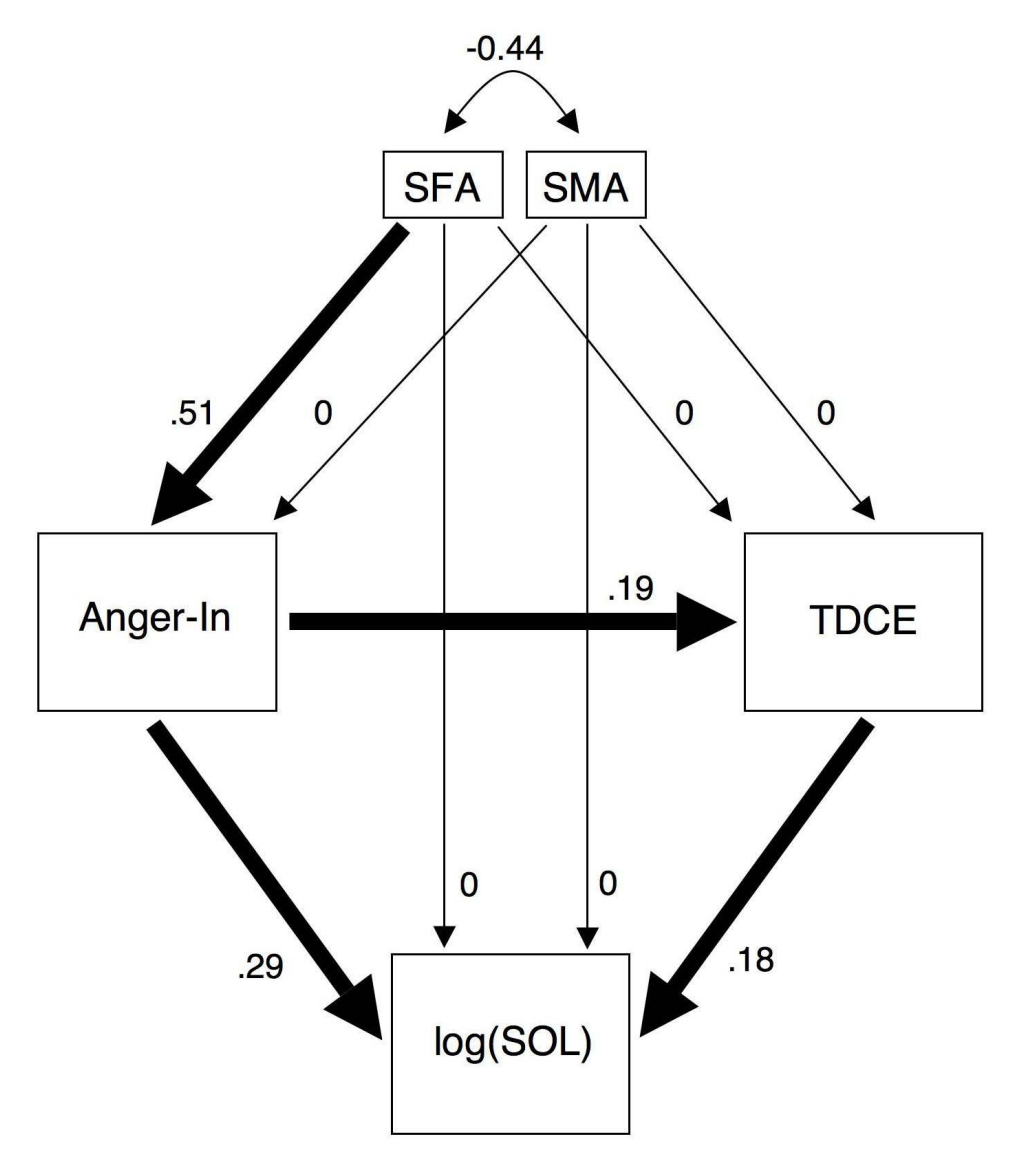
**Path diagram showing the relation between stereotypic feminine (SFA) and masculine (SMA) attribution, Anger-In, thermal discomfort from cold extremities (TDCE) and sleep onset latency [SOL, log transformed SOL (log(SOL)]**. Thick arrows indicate significant pathways (for details see text). Chi^2 ^= 5.151, df = 5 p = 0.398; CFI = 0.998; TLI = 0.995; RMSEA = 0.014; Pclose = 0.596.

To test how the variables combine together, a structure model was formulated with SOL as a purely endogenous variable, Anger-In and TDCE as partially endogenous variables and SFA and SMA as exogenous variables. Significant pathways (p < 0.05, indicated by thick arrows) are shown in Figure 2 with standardized path coefficients (partial regression coefficients) beside the path arrows. Such path coefficients are interpreted as in multiple regression models, thus controlling for the influence of other prior variables. The obtained model was recursive, over identified, and fitted the data well as shown by the different fit indices. Thus the chi-square fit index (Chi square = 5.151, df = 5, p = 0.398) clearly did not reach significance, which is a desirable result indicating that the model fit the data well. The three indices CFI (0.998), TLI (0.995) and RMSEA (0.014) indicated a very good fit. As assumed, there were in fact significant paths from SFA to Anger-In to TDCE to SOL. However, there is an additional direct path from Anger-In to SOL. No significant direct paths from SFA or SMA to SOL, and to TDCE were found and were therefore set to 0 in the model. When replacing SOL by 'minutes awake during nocturnal sleep' (an indicator for sleep maintenance), none of the two path coefficients leading to this new purely endogenous variable were significant, thus underlining the selectivity of our finding -- SOL seems to be sensitive to the influence of Anger-In, SFGS and TDCE.

## Discussion

The study shows that stereotypic feminine gender socialization, as indicated by high power-suppression, may constitute an important determinant for turning experienced anger inwards and subsequently leading to thermal discomfort with cold extremities and prolonged sleep onset latency.

From a sociological point of view, women can't be powerful because of their gender role - powerful women lose their femininity as it is defined and requested from society [28], p.143. In general, they occupy subordinate status in patriarchal societies that reserve power for men [29]. However, men may not necessarily get angrier than women. When women, and men, get angry it reflects a process of gender socialization, more specifically, how men and women have learned to cope with anger [27]. The high pressure to be a conformed woman in society can easily be observed in many behaviors as expressed in the widespread wearing of uncomfortable clothing and in typical feminine body positions (so-called gendering or doing gender, e.g. oblique head position, an attitude to submission and servility [30]), which do not allow expression of power.

In our sample we could show that women who rated themselves as more stereotypic feminine in comparison to other women (i.e. being less powerful) exhibit significantly higher anger suppression. Figure 1 depicts a kind of internal validation of gender power inventory using the validated instrument STAXI [19]. Women with high Anger-In rated significantly higher SFA than those with low Anger-In and conversely women with high Anger-Out rated higher SMA than those with low Anger-Out. Furthermore, gender differences in anger expression have to be expected from the obvious reality in the society. Diverse studies, including e.g. crime statistics [31], confirm that men as a sex are more physically and verbally aggressive than women. However, many studies using the validated instrument STAXI found no, or only small, gender differences in experiencing and expression of anger [19]. One reason for this discrepancy could be that women and men compare themselves within their own gender, abolishing therefore real existing differences. In other words, when the STAXI questionnaire would be filled out explicitly in comparison to the other gender, a clear gender difference can be expected (i.e. higher Anger-In in women and high Anger-Out in men).

The present study has limitations. First, it is a cross-sectional investigation and thus cannot causally assess the direction of the associations between the different dimensions (SFA, Anger-In, TDCE and SOL). To reveal a direct impact of e.g. SFA on Anger-In, prospective evaluations are required across different cultures and various age groups. Nevertheless, our analysis has confirmed what we a-priori hypothesized. Second, the study was based on self-reported information, which should be based on known validity and reliability of the defined scores. However, the chosen items for SFA and SMA loaded with low and high sex stereotype index scores of a former study using questionnaires concerning stereotypic gender socialization [22], respectively. This may at least indicate some external validity at least with respect to male or female associated sex-stereotype adjectives. Nevertheless, further validation studies are needed for SFA and SMA, which is presently under investigation. Additionally, in a separate sample of 25 women, we found good test-retest reliability for SFA and SMA over a two-week interval (r = 0.865 and r = 0.946, respectively). Internal consistency of the two scores has also been shown in the present study sample with a high Cronbach's alpha of 0.721 for the 10 SFA -items and 0.819 for the 11 SMA -items.

Moreover, for both physiological related measures (TDCE and SOL) external validation studies already exist. TDCE has been objectively validated by finger skin temperature measures [3] and SOL by polysomnographic recordings [32]. Additionally, ambulatory and controlled laboratory studies have shown that women with TDCE and prolonged SOL do, in fact, exhibit lower distal skin temperatures and longer SOL than controls [33,34]. It is well known that these physiological measures are strongly influenced by the autonomic nervous system (i.e. the sympathetic nerve activity) [2]. Chronic primary insomnia has been characterized as a state of hyperarousal seen, for example, in higher sympathetic nervous activity as measured by spectral analysis of heart rate variability [1,35]. We have recently shown that women with TDCE and difficulties initiating sleep exhibit a higher sympathetic/parasympathetic ratio found by heart rate variability analysis [36]. It can be assumed that activation of the sympathetic nervous system represents a biological pathway transforming anger suppression into manifested physiological changes such as TDCE and prolonged SOL. A recent study supports that anger suppression, and not outwardly expressed anger, is the significant determinant for somatoform disorders [37]. Our study suggests that sociocultural aspects, such as stereotypic gender socialization, have to be considered. It is well known that expression of anger and its somatization exhibit cultural differences. For instance, TDCE is even more prevalent in Japan (where it is called hi-e-sho, meaning 'cold syndrome' or 'vasospastic syndrome') than in European countries [9,38]. Taken together, in order to explain the chain of anger suppression to TDCE and prolonged SOL in women, SFGS has to be taken into account.

## List of abbreviations

AC: controlled anger expression; AI: anger expression inwards; AO: outwardly expressed anger; BMI: body mass index; CFI: comparative fit index; log (SOL): log transformed sleep onset latency; Pclose: p-value testing the null hypothesis that RMSEA is no greater than .05; RMSEA: root mean square error of approximation; SOL: sleep onset latency; SFGS: stereotypic feminine gender socialization; SFA: stereotypic feminine attributes; SMA: stereotypic male attributes; STAXI: state-trait-anger-expression-inventory; TDCE: thermal discomfort with cold extremities; TLI: Tucker-Lewis index

## Conclusion

Our findings suggest that stereotypic feminine gender socialization may play an important determinant for anger suppression, which subsequently can lead to thermal discomfort from cold extremities and prolonged sleep onset latency.

## Competing interests

The authors declare that they have no competing interests.

## Authors' contributions

MvA and KK conceived the study and wrote the manuscript. BG carried out the study and performed data processing. AHM and KK performed the data analysis. EZS, SO and JF provided advice on the study design and assisted in drafting the manuscript. All authors read and approved the final manuscript.
